# Osteoprotegerin in Cardiometabolic Disorders

**DOI:** 10.1155/2015/564934

**Published:** 2015-05-11

**Authors:** C. Pérez de Ciriza, A. Lawrie, N. Varo

**Affiliations:** ^1^Department of Clinical Chemistry, Clínica Universidad de Navarra, Avenida Pío XII 36, 31008 Pamplona, Spain; ^2^Department of Cardiovascular Science, University of Sheffield, Beech Hill Road, Sheffield S10 2RX, UK

## Abstract

Osteoprotegerin (OPG), a glycoprotein traditionally implicated in bone remodelling, has been recently related to cardiovascular disease (CVD). Human studies show a positive relationship between circulating OPG, vascular damage, and CVD, and as such OPG has emerged as a potential biomarker for CVD. This review focuses on the relationship between circulating OPG and different endocrine cardiometabolic alterations such as type 1 and 2 diabetes. The association of OPG with diabetic complications (neuropathy, nephropathy, or retinopathy) as well as with atherosclerosis, coronary artery calcification, morbidity, and mortality is pointed out. Moreover, OPG modulation by different treatments is also established. Besides, other associated diseases such as obesity, hypertension, and metabolic syndrome, which are known cardiovascular risk factors, are also considered.

## 1. Osteoprotegerin: Discovery and Structure

Osteoprotegerin (OPG) was first identified in 1997 simultaneously by two different research groups. Simonet et al. (1997) [1] were involved in a foetal rat intestine complementary deoxyribonucleic acid- (cDNA-) sequencing project when they discovered a new possible member of the tumour necrosis factor (TNF) receptor superfamily. It was named OPG because of its protective effects in bone (Latin: “*os*” bone and “*protegere*” to protect).

At the same time, another research group, Tsuda et al. [2], found a novel binding protein with no homology to known proteins in the conditioned medium of human embryonic lung fibroblasts which inhibited osteoclastogenesis. They termed this protein osteoclastogenesis inhibitory factor (OCIF). A year later, Yasuda et al. [3] published that these independent findings referred to the same molecule.

OPG/OCIF is a cytokine of the tumour necrosis factor (TNF) receptor superfamily [4], also termed as TNF receptor superfamily member 11B (TNFRS11B), and tropine reductase 1 [5]. The American Society of Bone and Mineral Research Committee has decided to use the term OPG as it implies its bone protective characteristics [6].

The OPG gene identified and cloned in 1998 [7] is a single-copy gene cluster on chromosome 8 (8q24) and it consists of five exons over 29 kilo bases (Kb) [8]. Northern blot analysis using a full-length cDNA probe produced three messenger ribonucleic acid (mRNA) transcripts of 2.4, 4.2, and 6.5 Kb [3]. The band at 2.4 Kb constituting the major transcript and the two other transcripts represent alternatively spliced forms containing all or a portion of the second intron that encodes for a soluble molecule [9].

Biochemically, OPG is a basic secretory glycoprotein composed of 401 aminoacids (Aa) that gives a monomeric weight of 60 kilodaltons (kDa). It has seven structural domains.

- Domains 1–4:* four cysteine rich pseudorepeats* structurally related to the TNF receptor family located in the N-terminal that is essential for the inhibition of osteoclastogenesis.
- Domains 5-6:* two death domains* at the carboxy-terminal end of the protein contain apoptosis-mediating death domain homologous regions.
- Domain 7: a* heparin binding site* is located in the C-terminal, capable of interacting with numerous proteoglycans as well as a free cysteine residue required for disulphide bond formation and dimerization [10, 11].

**Figure 1:**
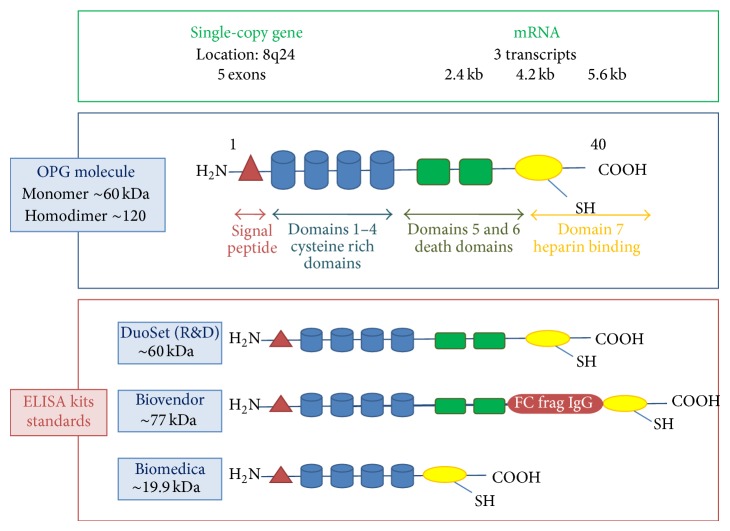
Osteoprotegerin structure and different ELISA kit standards.

In addition to its monomeric structure, OPG can be assembled at the cysteine 400 residue in the heparin binding domain to form a disulphide-linked dimer. Prior to secretion of both the monomeric and dimeric forms of OPG, the 21 Aa signal peptide is cleaved from the N-terminal rendering a 380 Aa mature OPG protein [12] (Figure 1). Thus, while the OPG monomer is biologically active, OPG homodimer form is more active and its formation is required to elicit full biological activity in vitro and in vivo [4, 9] because it possesses higher affinity for the receptor activator of nuclear factor-*κβ* (RANKL) ectodomain than the OPG monomer [11]. Tumour necrosis factor-related apoptosis-inducing ligand (TRAIL) and RANKL bind to OPG with similar affinities and they share common residues on OPG for their interaction [11].

OPG was initially described as an antiresorptive cytokine by binding principally to RANKL. However, since then, numerous ligands were described and studied giving OPG an increasing interest in vascular, tumour, and immune biology [11]. Clinical outcomes confirm that OPG is an active cytokine with potential use as a biomarker in a wide range of pathologies (osteoporosis, arthritis, vascular calcification, cancer bone-related disease, and so on) [11].

OPG is highly expressed in many different cell types such as osteoblasts, heart, kidney, liver, spleen, bone marrow [13], lung, thymus, lymph nodes, B-lymphocytes, articular chondrocytes [5], trachea, and testis. However, it is detected at very low levels in brain, placenta, and skeletal muscle [8].

In vitro studies indicate that OPG is expressed in cells involved in atheroma plaque development and progression, such as arterial smooth muscle cells [14] and pulmonary artery smooth muscle cells [15], in the Weibel-Palade bodies in endothelial cells [16] associated with von-Willebrand factor and in megakaryocytes [17] in the alpha granules. Moreover, OPG expression is enhanced in explanted human carotid atherosclerotic plaques [18]. Furthermore, OPG expression has recently been confirmed in human adipose tissue [19] (Table 1).

## 2. OPG/RANK/RANKL Pathway

The receptor activator of nuclear factor-*κβ* (RANK), another member of the TNF receptor superfamily, is a type I homotrimeric transmembrane protein consisting of 616 Aa including a signal peptide (28 Aa) with a 383-acid intracellular domain, a short transmembrane domain of 21 Aa, and a large C-terminal cytoplasmic domain [20, 21].

It is expressed on osteoclast precursors, mature osteoclasts, dendritic cells, B and T cells, fibroblasts, articular chondrocytes, and some cancer cells including breast and prostate cancers, tumours with very high bone metastasis potential [13]. After binding its ligand (RANKL), RANK assembles into functional trimeric receptor and this trimerization is required to generate multiple intracellular signals that regulate cell differentiation, function, and survival, among the other functional osteoclasts [22].

Receptor activator of nuclear factor-*κβ* ligand (RANKL) belongs also to the TNF superfamily and it is a type II homotrimeric glycoprotein consisting of 316 Aa, which exists as a transmembrane protein (40 to 45 KDa cellular form) and in a soluble form (31 KDa) [20]. Typically, RANKL is expressed and secreted by osteoblasts [5]. RANKL is also expressed in activated T-lymphocytes, lymph nodes, thymus, mammary glands, lungs, spleen, and bone marrow [13]. While OPG presents as a soluble bone protector, RANKL is considered to be a stimulator of bone resorption through the induction of osteoclasts' differentiation and activation of mature osteoclasts [22].

OPG seems also to play a key role on cell survival, via its interaction with tumour necrosis factor-related apoptosis-inducing ligand (TRAIL), another member of the TNF superfamily. TRAIL functions as a homotrimer and it is expressed as a type II transmembrane protein. The extracellular domain of this protein is proteolytically cleaved from the cell surface to act as a soluble cytokine [4].

**Figure 2:**
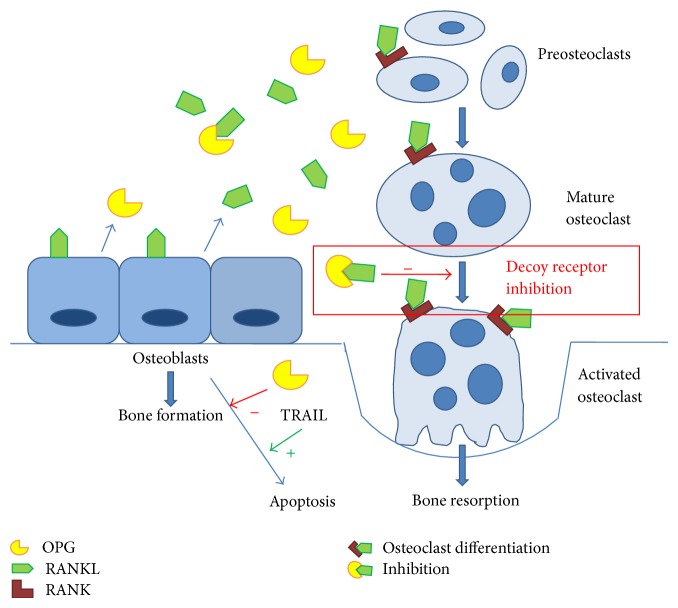
OPG/RANK/RANKL pathway.

Classically, the OPG/RANK/RANKL network is involved in bone remodelling and regulates the differentiation and activation of osteoclasts and hence the critical balance between bone formation and bone resorption. RANKL binds to RANK on osteoprogenitor cells and controls osteoclastogenesis and bone resorption. Initially, this RANKL-RANK interaction leads to the activation of nuclear factor-*κβ* that occurs by degradation of I*κ*B protein by I*κ*B kinase. This degradation of I*κ*B protein frees the nuclear factor-*κ*B complex, which then translocates to the nucleus initiating intracellular signalling cascades that lead to transcription of specific genes leading to osteoclast formation, differentiation, activation, and consequently bone resorption. OPG acts as a soluble decoy receptor, negatively regulating this interaction, and competes with RANK, inhibiting RANKL-RANK interactions [4, 23–26] (Figure 2).

## 3. Osteoprotegerin in Clinical Studies

**Figure 3:**
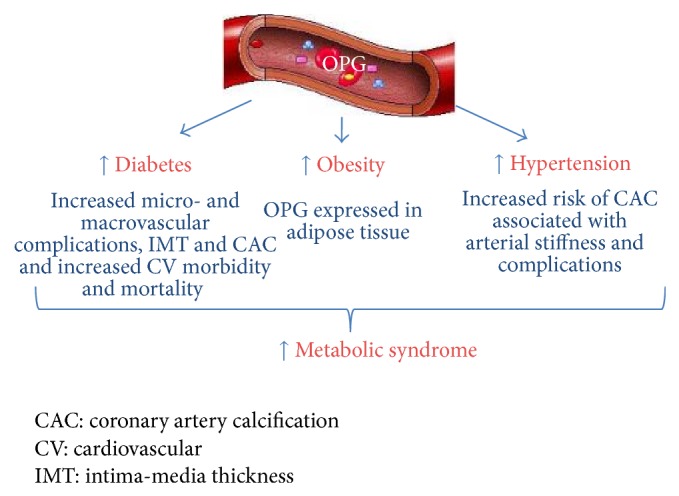
Summary of osteoprotegerin involvement in different endocrinological pathologies including diabetes, hypertension, obesity, and metabolic syndrome.

Although OPG has traditionally been implicated in bone remodelling and it has been determined as a biomarker in osteoporosis, the aim of this review is to highlight the association of OPG to other pathologies such as diabetes types 1 and 2, obesity, metabolic syndrome, or hypertension (Figure 3).

### 3.1. Diabetes Mellitus

In an animal model, Vaccarezza et al. [27] examined mRNA and protein OPG expression in control and streptozotocin-induced diabetic rats at early time points after the induction of diabetes. Diabetic rats showed a rapid and significant increase of the steady-state mRNA and protein levels of OPG in the aortic wall compared to control animals. Thus, an abnormal elevation of OPG in the vessel wall characterizes the early onset of diabetes mellitus and might represent a molecular mechanism involved in the vascular dysfunction characterizing this disease.

Different studies have shown that OPG levels are elevated in patients with type 1 diabetes mellitus (Table 2). Furthermore, prepubertal children with type 1 diabetes have significantly increased OPG levels [28]. Besides, Xiang et al. [29] also observed increased OPG in these patients. However, the study by Singh et al. showed opposite results in 35 patients with type 1 DM and in 25 age-, sex-, and ethnicity-matched healthy controls. Serum OPG levels were significantly lower in patients with type 1 DM compared to normal controls whereas RANKL levels were similar in both groups [30].

Different studies in type 1 diabetic cohorts have analysed the association between OPG and diabetic complications such as diabetic nephropathy [31–33] and neuropathy [34]. OPG is associated with poor glycaemic control and cardiovascular disease (CVD) in patients with type 1 diabetes, compatible with the hypothesis that OPG is associated with the development of diabetic vascular complications [31]. Besides, in another cohort of type 1 diabetic patients, not only OPG was elevated in patients with nephropathy but it also gradually increased with the severity (OPG levels were significantly elevated in patients with macroalbuminuria compared to microalbuminuria as compared with patients with normoalbuminuria and control subjects) [33]. This study highlighted the association between OPG and the presence and the severity of diabetic nephropathy. Besides, plasma OPG significantly correlated to peripheral neuropathy in type 1 DM, although differences in concentrations between both groups did not reach significance [34].

In addition to diabetic complications, OPG levels have also been associated with an increased risk of cardiovascular events as well as mortality [35–37]. In type 1 DM patients, OPG was associated with silent myocardial ischemia after correcting for other variables. The association of OPG with silent myocardial ischemia was observed in both genders, in type 1 and type 2 diabetic patients, in patients with or without nephropathy, and in patients without but not with peripheral arterial disease [37]. In a population study, OPG predicted an incident cardiovascular event and peripheral vascular disease/amputation events during follow-up (10.4 years) [35]. Furthermore, OPG was associated with mortality as well as with renal deterioration. Higher levels of OPG predicted all-cause and cardiovascular mortality in patients with diabetic nephropathy and deterioration of kidney function towards end-stage renal disease [36].

Different studies have also been performed in type 2 DM (Table 3) confirming that OPG levels were significantly higher in these patients compared to healthy controls [38–45]. Even after the exclusion of diabetic patients with a history of micro- or macrovascular disease, OPG levels remained significantly higher in diabetes [42] and in poorly controlled diabetic patients, serum OPG levels are higher than in well-controlled diabetic patients [46]. Moreover, OPG levels were increased in plasma from type 2 diabetic patients with microvascular complications [47, 48]. Furthermore, the association of OPG and diabetic nephropathy was also studied and OPG serum levels were significantly elevated in patients with microalbuminuria and macroalbuminuria as compared with patients with normoalbuminuria. In multivariate stepwise regression analysis, serum OPG has also been shown to be an independent factor associated with the severity of diabetic nephropathy [43]. Plasma OPG concentrations are significantly higher in type 2 DM patients with peripheral neuropathy [34], and serum and vitreous OPG concentrations were demonstrated to be higher in diabetic patients with retinopathy compared to those without [49]. In addition, plasma OPG concentrations positively correlated with diabetic neuropathy [44].

Type 2 DM is associated with increased atherosclerosis, with progressive vascular calcification being a major complication in the pathogenesis of this disease. Diabetic patients had higher mean intima-media thickness (IMT), a surrogate marker of atherosclerosis [50, 51], and OPG significantly associated with IMT [39]. Multivariate analyses revealed that the significant independent determinants of mean-IMT were age, hypertension, osteopontin, and OPG [50].

An emerging regulatory pathway for vascular calcification in diabetes involves the RANK, RANKL, and OPG [52]. Higher serum OPG levels are associated with higher prevalence of vascular calcification independently of progression of diabetic nephropathy [51, 53, 54]. Serum OPG level has also be demonstrated to be an independent predictor of vascular calcification and progression [54]. Moreover, OPG was significantly elevated in patients with increased coronary artery calcification (CAC). In multivariable analyses, OPG retained a strong association with elevated CAC scores after adjustment for age, gender, and other risk factors [55, 56]. In logistic regression analysis (after adjustment for age and main cardiovascular risk factors) serum OPG was associated with increased risk of abnormal IMT, carotid plaque, aortic calcification, and peripheral artery disease [51]. However, the study by Bourron et al. [57] suggested that although there was a significant association with OPG and calcification score in univariate analysis, it was no longer significant in multivariate analysis. RANKL and OPG/RANKL were not significantly associated with the calcification score.

Type 2 DM and increased OPG levels are associated with increased cardiovascular morbidity and mortality [52]. OPG was significantly associated with the presence [37] and severity of silent myocardial ischemia after adjustment for different risk factors [40]. There was an independent association of OPG with asymptomatic coronary artery disease in type 2 DM [58, 59]. Furthermore, increased plasma OPG concentration is associated with carotid and peripheral arterial disease in type 2 DM, whereas no relation is observed with myocardial ischemia [60]. Moreover, OPG levels were also higher in patients with high 10-year cardiovascular risk, patients with three or more damaged target organs, and patients with previous episodes of ischaemic cardiomyopathy or hypercholesterolaemia [38]. In a prospective observational follow-up study, elevated plasma OPG was a strong and independent predictor of all-cause mortality [61].

### 3.2. Osteoprotegerin in Insulin Resistance

Several studies have focused on the relationship between OPG and insulin resistance assessed by the homeostatic model assessment for insulin resistance (HOMA-IR). Yaturu et al. showed that OPG levels significantly correlated with insulin and insulin resistance [41]. Besides, O'Sullivan et al. [62] tested after a 75 g oral glucose tolerance test and OPG was higher in individuals with abnormal glucose tolerance but OPG did not correlate with HOMA-IR. Moreover, Hofsø et al. [63] demonstrated that oral glucose suppressed OPG levels, independently of obesity and glucose tolerance status indicating that glucose may be involved in the acute regulation of these proteins. Furthermore, the effect of acute hyperglycaemia on plasma levels of OPG in nondiabetic subjects was also studied. Acute hyperglycaemia increased plasma levels of OPG in nondiabetic subjects, whereas hyperinsulinaemia may suppress plasma OPG levels [64]. This observation is in accordance with Secchiero et al. [65] that showed that high glucose concentrations added to vascular endothelial cells did not modulate OPG release when used alone or in association with TNF-alpha.

Furthermore, insulin resistance and OPG levels have also been studied in obese individuals. Jorgensen et al. [66] studied the acute effects of insulin on plasma OPG in type 2 diabetic patients and obese individuals compared to lean controls. All subjects underwent a 4 h euglycemic-hyperinsulinemic clamp. Acute hyperinsulinemia decreased plasma OPG, but with diminished effect in individuals with type 2 DM and obesity. Besides, in obese adolescents, OPG levels and HOMA-IR index were significantly higher than in healthy volunteers and a significant positive correlation between OPG and insulin resistance was found [67]. However, in premenopausal obese and normal weight women the relationship between OPG and HOMA-IR was also assessed. Circulating OPG levels showed a negative and significant correlation with insulin and HOMA-IR [68].

In different healthy subjects (obese, overweight, or lean), OPG levels were negatively correlated with body weight, BMI, waist circumference, HOMA-IR, and fasting plasma insulin while being positively correlated with insulin sensitivity [69]. Ugur-Altun et al. [70] pointed out that insulin resistance in obesity is associated with decreased serum OPG levels. Moreover, a significant negative correlation was observed between OPG levels corrected for BMI and glucose, and insulin and HOMA-IR.

### 3.3. Obesity and Adipose Tissue

In the literature there is scarce and contradictory information regarding the relationship between OPG and obesity. Holecki et al. in obese perimenopausal women with concomitant diseases and obese general population found that serum OPG was significantly lower in comparison to normal weight controls [71]. However, Suliburska et al. [67] showed that serum OPG levels were significantly higher in obese adolescents than in controls. Furthermore, in a study by our group obese subjects exhibited increased OPG concentrations compared to nonobese subjects [19].

Abdominal adipose tissue is the largest fat tissue depot in the body and correlates with CV disease risk, MS, and other systemic inflammatory markers and may have an effect on atherosclerosis [72]. Adipose tissue releases several adipokines but also there is increasing evidence that there is a hormonal cross-link between adipose tissue and bone [73–75]. Interestingly, OPG expression has been recently confirmed in adipose tissue [19, 76–78]. In 2007, An et al. [76] described an increase in OPG expression during the differentiation process of 3T3L1, while there were no differences in RANKL expression. Besides, a higher OPG/RANKL ratio was observed after stimulation with TNF-*α* and a decrease in that ratio when cells were stimulated with insulin and rosiglitazone. Afterwards, Harsløf et al. [78] confirmed the expression of cytokines derived from bone in the adipose tissue including OPG. They observed an increased OPG expression with proinflammatory cytokines such as IL-1*β* and TNF-*α* and a reduced OPG expression when stimulated with cortisol or troglitazone. Besides, Fain et al. [77] in explants from human adipose tissue from obese women observed that OPG was secreted by both fat and nonfat cells. Moreover, Pérez de Ciriza et al. [19] confirmed increased OPG mRNA expression in adipose tissue from patients with the metabolic syndrome compared to healthy controls.

### 3.4. Metabolic Syndrome

The metabolic syndrome (MS) is a cluster of cardiometabolic alterations that include the presence of arterial hypertension, insulin resistance, dyslipemia, and abdominal obesity [79]. As explained before, individual criteria included in the MS definition such as DM, hypertension, and obesity have been shown to upregulate OPG concentrations. In the literature, there is scarce and contradictory information about the relationship between OPG and MS. Akinci et al. [80] showed that women with previous gestational DM (*n* = 46) developing MS had higher OPG levels than those without MS and healthy controls (*n* = 30). Furthermore, these results were confirmed in a larger cohort including 128 women with previous gestational DM and 67 age-matched controls. Serum OPG levels were associated with obesity, insulin resistance, and IMT [81]. However Nabipour et al. [82] did not find significant differences between the mean serum OPG levels of postmenopausal women with and without the MS. In a community-based study, Dallmeier et al. [83] observed a significant association between the MS and different inflammatory biomarkers except for OPG.

Recently, several publications have pointed out the positive relationship between OPG and MS. Pérez de Ciriza et al. [19] showed that OPG levels were significantly higher in MS patients compared to patients without the syndrome. Interestingly, OPG levels significantly and positively correlated with the number of cardiovascular risk factors. Besides, OPG expression in adipose tissue was confirmed and MS patients expressed higher OPG mRNA levels compared to those without. Bernardi et al. [84] concluded that OPG was elevated in patients with the MS compared to controls. Besides, in an animal model of MS (high-fat diet fed C57BL6 mice) they confirmed that OPG was elevated and that delivery of this protein promoted systemic and adipose tissue proinflammatory changes in association with metabolic abnormalities. They suggested that OPG may trigger adipose tissue proinflammatory changes in MS and high-fat diet induced obesity. Furthermore, in the study by Tavintharan et al. [47] higher OPG levels were associated with risk of MS and after adjusting for age, gender, ethnicity, glucose, and microvascular complications, OPG remained an independent predictor of MS.

### 3.5. Hypertension

OPG levels have also been related to hypertension. Stępień et al. [85] showed that OPG levels were significantly elevated in hypertensive subjects (*n* = 130) compared to normotensives. Furthermore, multiple regression analysis demonstrated that inflammation, age, and hypertension were predictors of increased OPG levels. Furthermore, in a study by our group [19] OPG concentration was also significantly higher in hypertensive subjects confirming the results by Stepien. OPG levels were associated with increased risk of coronary calcification in asymptomatic normotensive individuals, and renal function significantly contributed to this process in both hypertensive and normotensive subjects [86].

Significant correlations were found between OPG levels and age, height, glycaemia, systolic, diastolic, and pulse blood pressure, pulse wave velocity, and left ventricular hypertrophy in hypertensive patients. In hypertensive subjects, markers of inflammation are elevated and the pressure of arterial blood may stimulate the endothelium promoting the inflammatory cascade and increasing OPG concentration. Increased OPG concentrations were also reported in hypertensive patients suffering from related complications [38]. The serum OPG level is positively associated with arterial stiffness in hypertensive patients. In addition, a multivariate logistic regression analysis showed that age, diastolic blood pressure, and OPG levels were independent predictors of arterial stiffness in the hypertensive patients [87].

## 4. Polymorphisms of the Osteoprotegerin Gene

Different single nucleotide polymorphisms (SNPs) known to be associated with osteoporosis (T245G, T950C, and G1181C) were evaluated in type 2 DM to study whether they contributed to CVD in these patients. The C allele of the T950C polymorphism was independently associated with higher risk of CVD in type 2 DM. However, there was no significant association between the T245G and G1181C polymorphisms and CVD [88].

Besides, the association of diabetic retinopathy and two different SNPs of the OPG gene, rs2073618 (located in exon I) and rs3134069 (located in the promoter region), were also studied. Logistic regression analysis demonstrated that the carriers of the CC genotype had a 2.2 higher risk for diabetic retinopathy than those with either the CG genotype or the GG genotype (codominant model for rs2073618). Furthermore, the combined effect of SNPs on the diabetic retinopathy was stronger than that of each SNP alone. These results indicate that SNPs in the OPG gene may be involved in the pathogenesis of diabetic retinopathy [89].

Finally, the association of rs2073617, rs2073618, and rs3134069 SNPs and diabetic foot was evaluated. The A allele of the rs2073617 polymorphism protected women in variant AA versus AG against diabetic foot compared with controls. Besides, in the rs2073618 polymorphism, the C allele was a risk factor for diabetic foot. However, the rs3134069 polymorphism was not observed to be a risk factor for diabetic foot [90].

Different SNPs are also related to blood pressure or hypertension. In a large cohort of elderly men, the SNP, rs11573901, was significantly associated with diastolic blood pressure, after adjusting for other risk factors. Men with the TC genotype had lower diastolic blood pressure than those with the common CC variation. However, this SNP was not associated with plasma OPG in the population examined [91].

The SNP in the promoter region of OPG (T950C) was associated with vascular morphology and function in healthy individuals [92]. The SNP was then studied in another larger population of patients with hypertension and left ventricular hypertrophy. Hypertensive subjects with the CC genotype showed significantly increased IMT compared to those hypertensives with the TC and TT genotypes. The allele distribution did not differ between hypertensive and control individuals. This study showed that SNP in the promoter region of OPG is associated with vascular morphology in hypertensive subjects [93].

## 5. Therapeutic Interventions That Modulate OPG

Several treatments are known to affect OPG concentrations such as insulin, glitazones, and statins. OPG levels significantly decreased in both type 1 [29] and type 2 [94] DM, after six months of insulin treatment. Besides, in type 2 DM (*n* = 67), pioglitazone (15 * *mg/day, *n* = 34) treatment for six months decreased OPG levels significantly. However, metformin treatment (1000* * mg/day, *n* = 33) for the same time did not vary OPG concentration [95]. Furthermore, in patients from the South Danish Diabetes Study (*n* = 371), rosiglitazone treatment for 2 years caused a significant decrease in plasma OPG concentrations while treatment with metformin or insulin did not change OPG [96].

Furthermore, treatment of hypercholesterolemic type 2 DM with different statins showed contradictory results. Simvastatin reduced plasma OPG levels [97] while OPG was increased after pravastatin [98] and lovastatin treatment [99]. The withdrawal of lovastatin decreased serum OPG level [99].

However, in patients that had never received statins before, statins decreased OPG levels. Simvastatin [100] and atorvastatin (20 mg/day) [101] treatment significantly decreased serum levels of OPG. Besides, patients with hypertension (*n* = 48) were treated with 20 mg olmesartan combined to 16 mg azelnidipine or 1 mg indapamide. Azelnidipine, but not indapamide, combined with olmesartan improved arterial stiffness and was associated with significant decrease in OPG [102].

In addition to different treatment, other interventions modulate OPG concentration. Weight reduction therapy resulted in a further decrease in OPG serum concentrations [103]. However, bariatric surgery did not modify OPG levels although there was an improvement in other parameters [104]. Besides, in overweight and obese patients undertaking a 6-month exercise programme, OPG levels did not change significantly [105].

## 6. Pathophysiological Role of Osteoprotegerin

Nowadays there is emerging evidence of the role of OPG in the pathogenesis of atherosclerosis, calcification, and CVD. Different studies have highlighted different potential mechanisms that may explain the association. Evidence is accumulating that OPG may be expressed, be regulated, and function in vascular physiology and pathology in unique ways to promote endothelial cell survival, angiogenesis, monocyte, or endothelial cell recruitment, and smooth muscle cell osteogenesis, and calcification [106].

In endothelial cells, OPG acted as a survival and antiapoptotic factor. OPG protected endothelial cells from apoptosis in vitro and promoted neovascularization in vivo. Besides, OPG increased endothelial cell proliferation in microvessels [107]. Moreover, OPG stimulated the expression of intercellular adhesion molecule-1, vascular cell adhesion molecule-1, and E-selectin by endothelial cells in the presence of TNF-alpha [108]. Furthermore, emerging evidence suggests a role of OPG in endothelial cell survival and treatment of endothelial cells with proinflammatory cytokines, which secreted OPG to the supernatant [16].

In addition to its effects on endothelial cells, OPG increased the expression of adhesion molecules as well as monocyte binding to endothelial cells [108]. Furthermore, in an in vivo model, Zauli et al. [109] demonstrated that leukocyte/endothelial cell adhesion and leukocyte rolling was promoted by OPG. Moreover, in plaques from different locations, OPG expression by staining was correlated with the abundance of macrophages in the lesions [110].

**Figure 4:**
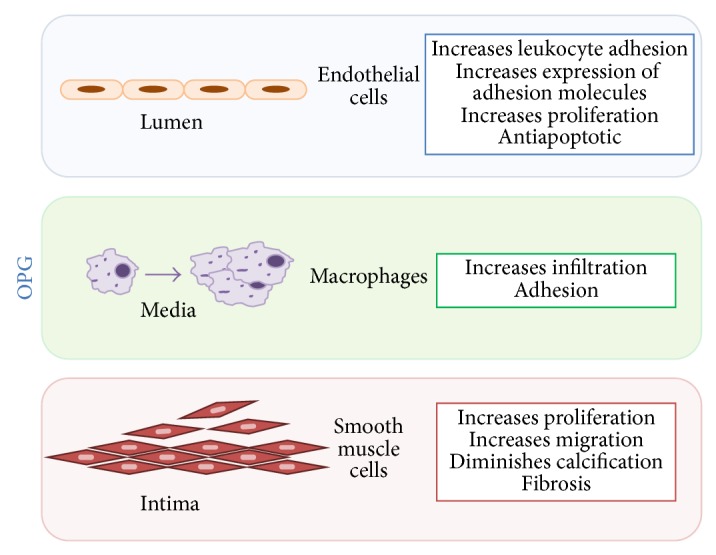
Pathological role of osteoprotegerin in endothelial cells, smooth muscle cells, and macrophages.

In vascular smooth muscle cells, both in vitro and in vivo treatments using OPG induced signs of fibrosis [111] and inhibited vascular calcification [112]. Moreover, recombinant OPG promoted vascular smooth muscle cells proliferation in both human [15] and rodent cells [113]. All these findings suggest that OPG may be involved in different processes that lead to atherosclerosis and CVD (Figure 4).

In spite of this evidence of OPG as a proatherogenic mediator and the association of OPG with atherosclerosis risk factors, discussion still exists on the meaning of OPG as a marker of a mediator of cardiovascular disease.

Some hypotheses suggest that the association of increased OPG seen in cardiovascular disease is the result of an incomplete compensatory mechanism. It is possible that circulating OPG levels are increased in response to the initial vascular insult and ongoing process of inflammation within an atherosclerotic plaque lesion as the component of a complex compensatory mechanism [4]. Indeed, recent evidence suggests that damaged endothelial cells may release OPG from Weibel-Palade bodies in response to inflammation, thus increasing circulating levels. Although vascular endothelial cells may be the source of the circulating OPG with the onset of atherosclerosis, it is not clear whether increased expression is required for the changes in circulating levels [114]. Besides, in diabetic patient, increased OPG production may represent an early event in the natural history of diabetes mellitus, possibly contributing to disease-associated endothelial cell dysfunction [65].

However, different findings in animals [114] suggest that endogenous OPG may be a marker, rather than a mediator, of atherosclerosis. Furthermore, OPG elevation appears to be a marker of atherosclerosis onset rather than its severity or progression.

On the other hand, various studies highlight the apparent proatherogenic role of increased OPG itself taking part in different steps involved in the atherogenic process. It seems that OPG plays a role in endothelial cell stimulation and dysfunction [16, 108] as well as macrophage infiltration [110] and leukocyte adhesion [109]. Moreover, OPG promotes SMCs proliferation [15, 113]. Circulating OPG levels could probably indicate ongoing EC injury as well as activation of the SMCs, which have been observed in progressing plaque lesions. An increased OPG level could be an indicator of a proinflammatory milieu responsible for propagation of atherosclerosis [4].

OPG would appear to have a dichotomous role in humans. In healthy individuals, the proatherogenic and antiatherogenic effects are being held in a fine balance, but in the face of persistent positive induction by various risk factors the proatherogenic pathway becomes predominant to the detriment of the subject [4]. Besides, the role of OPG differs between studies in humans and animals. Observational studies in humans show a positive relationship between serum OPG levels and clinical cardiovascular disease whereas animal studies support a protective role for OPG [114].

Thus, more research is required to elucidate whether OPG is an ineffective marker of CVD or if it represents a pathogenic factor.

## 7. Osteoprotegerin as a Biomarker

OPG is highly expressed in both bone and vasculature. Although several studies and different evidence suggest the involvement of OPG in cardiovascular risk and cardiovascular disease, more evidence is needed to evaluate the predictive and diagnostic value of serum OPG levels for clinical use as well as its pathogenic importance [115].

In view of the association between OPG and different cardiovascular risk factors in humans, there is significant interest in developing OPG as a biomarker. Its clinical role is limited due to the expression by numerous types of tissues in vivo. Identifying tissue isoforms will potentially increase the clinical utility as a biomarker. Furthermore, OPG levels measured in circulation correspond to the OPG monomer, dimer, and bound OPG. As a result it is necessary to better define and establish OPG measurement conditions and the potential sources of variability.

In order to implement OPG as a biomarker in the clinical laboratory setting, it is important to consider different preanalytical and analytical variables that may influence its measurement.

OPG is measured using a variety of ELISA kits with different standards with different molecular weights resulting in differences in the final OPG concentration as highlighted by Clancy et al. [116]. These variations appear largely attributable to differences in the standards used. R&D DuoSet standard is similar to full-length OPG suggesting that this ELISA kit may be more representative of the OPG molecule (Figure 1). Besides, as suggested by Naylor et al. [117] OPG exists in different forms in serum, monomer, and dimmers, and the cross-reactivity of available immunoassays to these components is unknown.

Circulating OPG can be measured in both plasma and serum. However, the resultant concentration is not comparable due to the lower OPG levels observed in serum samples and higher OPG levels in all plasma samples [118] including EDTA [119]. Due to this, caution should be taken when comparing OPG concentration from different studies due to the differences observed among sample type and standards used. Besides, each laboratory should determine adequate reference ranges for each specimen. Furthermore, circulating OPG reflects the production from several tissues, which makes it difficult to specify the site of origin [120].

After blood collection, other steps such as transport, delayed processing, centrifugation, or storage should be adequately controlled. Increasing centrifugation forces do not alter OPG concentration and serum separation after centrifugation should be performed as soon as possible (within 30 minutes). Serum centrifuged samples are more stable and they should preferably be stored at 4°C for a maximum period of 24 hours. Longer storage may be done at −20°C [118] or at −70°C [119]. OPG concentration increased after several freeze-thaw cycles [118, 121]; a maximum of three is suggested in order to minimize this increase.

Although the clinical prognostic utility of OPG seems to be awhile away yet, considering these recommendations, OPG quantification may be more stable and reproducible. Quantifying OPG holds a great deal of promise in helping the clinician risk to stratify patients with cardiovascular disease more accurately in combination with other markers to provide clinically relevant information. However, more research is necessary in order to better clarify the role of OPG in cardiovascular disease.

## 8. Conclusion

OPG levels have been related to different cardiometabolic alterations such as diabetes, obesity, hypertension, and metabolic syndrome. Furthermore, increased OPG levels associate with poor diabetes control, diabetic complications, atherosclerosis, CAC, and mortality. As a result, OPG may be a potential biomarker of complications and severity. However, more evidence is needed to evaluate the predictive and diagnostic value of serum OPG levels for clinical use as well as the possible mechanism involved in the increase observed. Furthermore, implementation of OPG determination in the clinical laboratory setting would be useful in order to better stratify patients and to assess the most adequate treatment. Nevertheless, more research and more evidence will be needed in order fully assess OPG usefulness in the laboratory as a biomarker.

## Figures and Tables

**Table 1:** Osteoprotegerin expression and regulation in different tissues.

Cell type	OPG expression	Cytokine	Reference
Osteoblasts	Upregulation	IL-1*α*, IL-6, IL-11, IL-18, TNF-*α*, bone morphogenic protein type 2 (BMP-2), calcium, vitamin D3, estrogens, angiotensin II and platelet derived growth factor (PDGF), TNF-*β*, IL-1*β*, transforming growth factor *β* (TGF-*β*), 17-estradiol and Wnt signalling pathway	[5, 119]
	Dowregulation	Parathyroid hormone (PTH), glucocorticoids, prostaglandin E2, calcium, immunosuppressant drugs, peroxisome proliferators activated receptor (PPAR-*γ*), basic fibroblast growth factor (bFGF)	[5, 119]

Endothelial Cells	Upregulation	IL-1*α*, IL-1*β* and TNF-*α*	[16]

Smooth Muscle Cells	Upregulation Dowregulation	IL-1*β*, TNF-*α*, estrogen and PDGF TGF-*β* and BMP-2	[14]

Bone Marrow Stromal Cells	Upregulation Dowregulation	IL-18, TNF-*α*, BMP-4 and TGF-*β* IGF-1, IL-6, IL-11, IL-17 and IL-1 *β*	[5, 119]

Dendritic Cells	Upregulation	TNF-*α*, RANKL, IL-1 *β* and CD40 ligand	[5, 119]

Megakariocytes			[17]

Adipose Tissue	Upregulation Dowregulation	IL1-*β* and TNF-*α* Cortisol or troglitazone	[19, 76–78]

Other tissues: heart, kidney, liver, spleen, lung, thymus, lymph nodes, B-lymphocytes, articular chondrocytes, trachea and testis. Very low levels in brain, placenta and skeletal muscle			[5, 8, 13]

**Table 2:** Studies assessing the association between osteoprotegerin and diabetes mellitus type 1.

Author, Date	Population	Findings
Xiang et al., 2007 [29]	22 newly diagnosed type 1 DM 28 healthy subjects	Plasma OPG levels are elevated in newly diagnosed type 1 DM
Singh et al., 2010 [30]	35 type 1 DM 25 sex, age, ethnicity matched controls	Serum OPG levels were significantly lower in patients with type 1 DM compared to normal controls.
Rasmussen et al., 2006 [31]	199 type 1 DM without diabetic nephropathy 192 type 1 DM with diabetic nephropathy	OPG associated with cardiovascular disease and glycaemic control
Grauslund et al., 2010 [32]	Population-based Fyn County Denmark 200 type 1 DM long diabetes duration	OPG associated with higher risk of nephropathy
Wang et al., 2013 [33]	80 type 1 DM 30 controls	OPG associated with the presence and severity of nephropathy
Nybo et al., 2010 [34]	200 type 1 DM 305 type 2 DM	OPG correlated with diabetic neuropathy
Avignon et al., 2007 [37]	465 diabetic patients with one additional risk factor	OPG is associated with silent myocardial ischemia
Gordin et al., 2013 [35]	1,939 adults (population FinnDiane) Finnish Diabetic Nephropathy Study	OPG predicted an incident cardiovascular event and peripheral vascular disease Follow-up 10.4 years
Jorsal et al., 2008 [36]	397 type 1 DM overt nephropathy 176 type 1 DM with persistent normoalbuminuria	OPG associated with mortality and renal deterioration Follow-up 11.3 years. Prospective observational study

**Table 3:** Studies assessing the association between osteoprotegerin and diabetes mellitus type 2.

Author, Date	Population	Findings
O'Sullivan et al., 2010 [42]	62 type 2 DM 58 healthy subjects (age, gender, BMI)	OPG is significantly increased in type 2 DM
Altinova et al., 2011 [46]	166 type 2 DM	OPG levels are higher in poorly controlled DM
Yaturu et al., 2008 [41]	50 type 2 DM 59 subjects without DM	OPG is elevated in type 2 DM and correlated with insulin resistance. Cross-sectional study
Knudsen et al., 2003 [48]	80 subjects divided in four groups according to glucose tolerance	OPG is increased in patients with microvascular complications
Chang et al., 2011 [43]	179 type 2 DM	OPG is associated with diabetic nephropathy
Xiang et al., 2007 [29]	154 newly diagnosed type 2 DM 96 healthy controls	Plasma OPG levels are associated with urinary albumin excretion
Nybo et al., 2010 [34]	200 type 1 DM 305 type 2 DM	OPG is increased in patients with peripheral neuropathy
Yu et al., 2015 [49]	254 diabetic patients (100 without retinopathy, 154 with retinopathy), 62 controls	OPG is associated with retinopathy. Patients with retinopathy exhibit increased vitreous and serum OPG
Terekeci et al., 2009 [44]	42 type 2 DM 24 healthy controls	OPG correlated with diabetic neuropathy
Tavintharan et al., 2014 [47]	1,220 type 2 DM	Higher OPG levels associated with microvascular complications (nephropathy, neuropathy and retinopathy)
Ishiyama et al., 2009 [50]	168 type 2 DM 40 non-diabetic subjects	Diabetic patients had higher IMT and OPG was an independent determinant of IMT
Moreno et al., 2013 [51]	68 males (43 with type 2 DM and 25 subjects without diabetes)	Serum OPG associated with increased risk of abnormal IMT, carotid plaque, aortic calcification and peripheral disease
Gaudio et al., 2014 [39]	40 type 2 DM postmenopausal women 40 healthy controls	OPG associated with IMT
Singh et al., 2012 [54]	58 type 2 DM	OPG was an independent predictor of baseline vascular calcification and progression
Anand et al., 2006 [55]	510 type 2 DM	OPG is elevated in patients with CAC
Anand et al., 2007 [56]	398 type 2 DM	OPG associated with elevated CAC
Bourron et al., 2014 [57]	198 type 2 DM	LnOPG associated with calcification score. Cross-sectional study
Avignon et al., 2007 [37]	645 diabetic patients with one additional risk factor	OPG associated with the presence of silent myocardial ischemia
Guzel et al., 2013 [40]	45 type 2 DM 33 healthy controls	OPG associated with the presence and severity of silent myocardial ischemia
Reinhard et al., 2011 [58]	200 asymptomatic DM patients without known cardiac disease	OPG independently associated with asymptomatic coronary artery disease
Avignon et al., 2005 [59]	162 asymptomatic type 2 DM	OPG independently associated with asymptomatic coronary artery disease
Poulsen et al., 2011 [60]	305 type 2 DM without known cardiovascular disease	Increased plasma OPG associated with carotid and peripheral arterial disease. No association with myocardial ischemia
Blázquez-Medela et al., 2012 [38]	52 type 2 patients 54 healthy controls	Higher OPG levels were observed in patients with higher probability of 10-year cardiovascular risk
Reinhard et al., 2010 [61]	238 type 2 DM	Elevated plasma OPG was a strong predictor of all-cause mortality. Follow-up 16.8 years. Prospective observational study

## Abbreviations

Aa: Aminoacid
BMI: Body mass index
CAC: Coronary artery calcification
CVD: Cardiovascular disease
DM: Diabetes mellitus
ELISA: Enzyme-linked immunosorbent assay
HOMA-IR: Homeostatic model assessment for insulin resistance
IMT: Intima-media thickness
Kb: Kilobases
mRNA: Messenger ribonucleic acid
MS: Metabolic syndrome
OCIF: Osteoclastogenesis inhibitory factor
OPG: Osteoprotegerin
RANK: Receptor activator of NF-*κβ*
RANKL: Receptor activator of NF-*κβ* ligand
SNP: Single nucleotide polymorphism
TNF: Tumour necrosis factor
TRAIL: Tumour necrosis factor-related apoptosis-inducing ligand.
